# In vivo non-contact regions of proximal scaphoid in six extreme wrist positions

**DOI:** 10.1186/s12891-024-07561-4

**Published:** 2024-06-06

**Authors:** Ren-Guo Xie

**Affiliations:** 1 Department of Hand Surgery, Shanghai General Hospital, Shanghai Jiao Tong University, School of Medicine, 650 New Songjiang Road, Songjiang, Shanghai 201620 China; 2 Department of Hand Surgery, Affiliated Hospital of Nantong University, 20 West Temple Road, Nantong, Jiangsu 226001 China

**Keywords:** Non-contact regions, Scaphoid, Wrist position, In vivo

## Abstract

**Introduction:**

Fractures of the scaphoid are the most common carpal injuries, account for 80-90% of all carpal fractures. 5-15% nonunion of scaphoid fractures were reported even with adequate primary treatment, which probably progresses to osteoarthritic changes several decades later. Researches regarding to scaphoid physiological characteristic in vitro and in vivo and kinds of trials in clinical practice are being kept on going, which contribute much to our clinical practice. With the advancing wrist arthroscopy, 3D-print patient-specific drill guide, and intraoperative fluoroscopic guidance, dorsal approach (mini-invasive and percutaneous technique) is being popular, through which we can implant the screw in good coincidence with biomechanics and with less disturbing tenuous blood supply of the scaphoid. Investigating the noncontact area of the dorsal proximal scaphoid in different wrist positions can facilitate preoperatively estimating insert point of the screw.

**Materials and methods:**

Eight volunteers were recruited to accept CT scans in six extreme wrist positions. The images of DICOM mode were imput into the Mimics analytical system, the segmented scaphoid, lunate and radius were exported in mode of ASCII STL and were opened in the software of Geomagic studio. We created four planes based on anatomic markers on the surface of the radius and scaphoid to confine the proximal scaphoid to form the so-called non-contact regions. We measured and compared the areas in six targeted positions.

**Results:**

Amidst six extreme wrist positions, area of the non-contact region in extreme dorsal extension (59.81 ± 26.46 mm^2^) was significantly the smallest, and it in extreme palmar flexion significantly was largest (170.51 ± 30.44 mm^2^). The non-contact regions increased in order of dorsal extension, supination, ulnar deviation, radial deviation, pronation and palmar flexion. As for two-group comparison, the non-contact region showed significantly larger (*p* < 0.05) in palmar flexion than the others except for in pronation individually, and in radial deviation (*p* < 0.05) than in dorsal extension.

**Conclusions:**

Sufficient space was available for the screw started from the dorsal approach despite the wrist positions.

## Introduction

The cartilage covers most of the scaphoid, accounting for 80% of its surface, and composes four articular facets, which results in scaphoid fractures could almost entirely be considered as the intra-articular fractures [1, 2]. With minimal soft-tissue and no periosteum attachments, the fractured scaphoid healing type is primary bone heal, a kind of so-called “cutting cone” forming and remodeling. For this specific healing mode of the scaphoid, rigid internal fixation with compression to eliminate the intra-fragmental gap and mobilization would seemly be mandatory [3, 4]. Screw plays a major role in the implant for the broken scaphoid, while there is technically demanding much difficult in plates [5, 6]. Dodds et al. demonstrated the longest possible screw placed centrally and deep in both the proximal and distal scaphoid poles would have biomechanical superiority in for the fracture fixation stabity [7]. McCallister et al. conducted a single load-to failure experiment in fresh cadaveric specimens and concluded that the ideal screw placement was in the central zone along the long axis of the scaphoid [8]. Two-screw fixation showed theoretically and pragmatically stable properties with resistance to any displacement [9–11]. In practice, moreover physicians should have to evaluate the patient-specific morphological classification and facture location to get the optimal pre-operative plan. In the open reduction and internal fixation, volar approach for screw implant impeded with the trapezium and dorsal approach would maim the tenuous vascular supply. With the arthroscopy gradually widely evolved and the three-dimensional printed guide newly assisted in wrist surgery [12], mini-invasive and percutaneous screw introduced through dorsal approach would be more and more practicable. While the screw was implanted for the scaphoid fracture, protrusion of its head and tip commonly occurred [13], which might result in cartilage injury. Sometimes there found osteocartilaginous outgrowth of about 1 mm over the entry point, during the screw removal. Although no affirmative evident, these cartilage malady around the entry point in joint contact areas might cause osteoarthritis. Here we measured the non-contact area of proximal scaphoid in six extreme wrist positions, which could yield some information available for starting point of the screw.

## Materials and methods

Six hand surgeons and two radiologists took part in this investigation as the volunteers, aged from 28 years to 45 years, weighted from 69 kg to 82 kg, heighted from 170 cm to 180 cm, and all were male. Our hospital review board approved this study (2019KY166). Clinical examinations didn’t invoke any symptom and any impediment of the carpal motion, posteroanterior and lateral radiographs didn’t show any abnormal skeletons, and no any remarkable disorder history were recalled in their upper limbs.

A computed tomography (CT) scanner (SOMATOM Definition Flash, Siemens Medical, Forchheim, Germany) was used to obtain the volume images for the volunteers’ wrists at tube setting of a maximum of 120 kVp and 100 mA and a slice thickness of 0.6 mm.

Volunteers were positioned prone on the CT table, with the right arm stretched over the head, the shoulder abducted slightly and flexed at about 120 degrees, the elbow flexed slightly, and the wrist centered in gantry. This position was kept with several pillows under the volunteer’s chest and arm, making him comfortable and only his wrist no constrained during the CT scanning.

Subjects actively moved their wrist to six extreme positions and kept them, flexion, extension, radial deviation, ulnar deviation, pronation and supination. Contiguous scanning was performed from the distal part of the radius and ulna to the middle part of the metacarpus. Each position was scanned independently for 7 s.

We used Mimics 21.0 (Materalise, Leuven, Belgium) to segment and to reconstruct the 3-dimensional structures of two-row carpals, the distal part of the radius and ulna, and the third metacarpus, which could facilitate to reassure the wrist positions. Then we anatomically block- segmented and exported the distal radius and the scaphoid in form of ASCII (American Standard Code for Information Interchange) STL (Stereolithography).

**Fig. 1:**
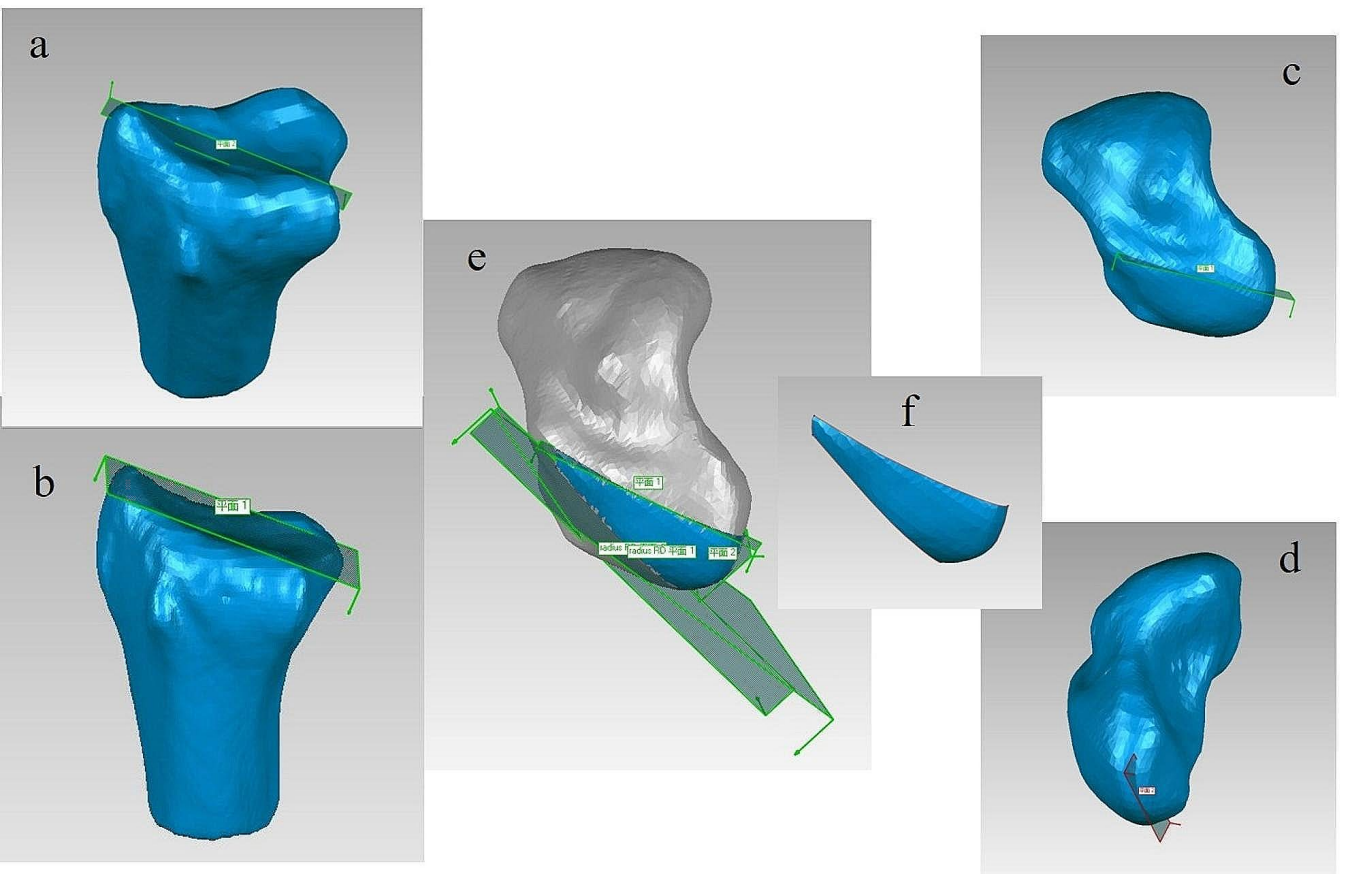
Four planes (**e**) were created to confine proximal part of the scaphoid into the non-contact region (**f**). Plane one (**a**) was perpendicular to the articular aspect of the distal radius and included two points, the tip of radial styloid and the dorsal tip of the radial sigmoid notch. Plane two (**b**) was based three points, the tip of radial styloid and the dorsal tip and the volar tip of the radial sigmoid notch. Plane three (**c**) was decided with three points along the proximal ridge of the scaphoid. Plane four (**d**) was paralleled with the scapholunate interosseous conjuncture through the inflection points of the scaphoid (from the proximal part to the scapholunate interosseous conjuncture)

The aforementioned file was opened in a reverse-engineering software (Geomagic Studio 2013, Geomagic Inc., North Carolina, USA). We created four planes, within which the confined proximal part of the scaphoid was considered as the non-contact region (Fig. 1). P lane one was perpendicular to the articular aspect of the distal radius and included two points, the tip of radial styloid and the dorsal tip of the radial sigmoid notch. Plane two was based three points, the tip of radial styloid and the dorsal tip and the volar tip of the radial sigmoid notch. Plane three was decided with three points along the proximal ridge of the scaphoid. Plane four was paralleled with the scapholunate interosseous conjuncture through the inflection points of the scaphoid (from the proximal part to the scapholunate interosseous conjuncture). We calculate areas of the whole scaphoid surface and the non-contact regions.

Area of the non-contact regions for each targeted position and its percentage in the whole scaphoid surface were shown as average ± 1 standard deviation. Distribution of the data was evaluated with the one-way analysis of variance procedure. Post hoc, Student-Newman-Keuls pairwise comparison method was used to obtain the significance of the differences between every two positions. The level of significance was set at 0.05 and a statistical power should not be less than 0.80.

## Results

**Fig. 2:**
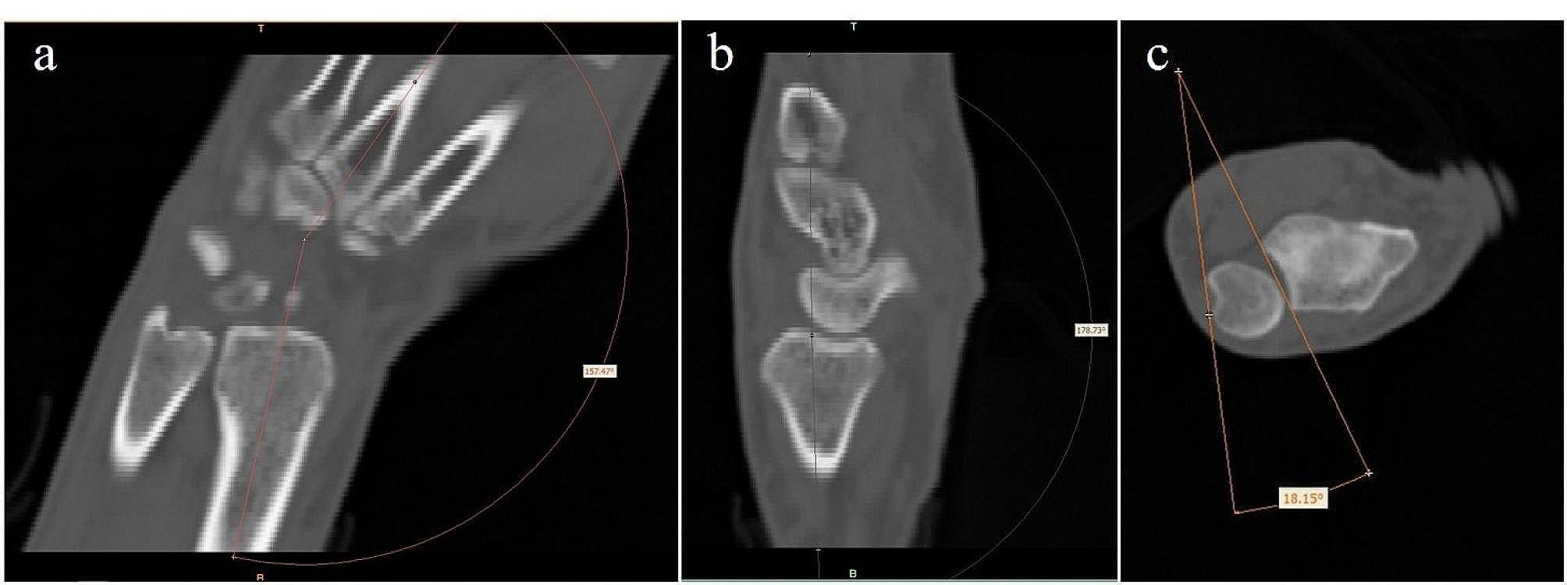
In the CT images, longitudinal axes of the third metacarpus and of the distal radius in the coronal plane and in the sagittal plane forming the angles of the radio-ulnar deviation (**a**) and the flexion-extension (**b**), respectively, and lines connecting two tips of the radial sigmoid notch and of the ulnar ECU (extensor carpi ulnaris tendon) notch in the horizontal plane forming the angles of rotation (**c**)

We didn’t use customized stabilizing devices for the targeted positions, in which there were three specific angles for each subject (Table 1), and we measured the value of these angels in the CT images as illustrated in Fig. 2, longitudinal axes of the third metacarpus and of the distal radius in the coronal plane and in the sagittal plane forming the angles of the flexion-extension and the radio-ulnar deviation, respectively, and lines connecting two tips of the radial sigmoid notch and of the ulnar ECU (extensor carpi ulnaris tendon) notch in the horizontal plane forming the angles of rotation. The extreme extension and flexion could be accompanied with two slight different positions (radial or ulnar deviation, pronation or supination) other than with the neutral, and so on for the left four extreme positions (radial deviation, ulnar deviation, pronation and supination), although all subjects with professional qualifications was taught and elucidated to obtain the targeted positions. All targeted positions, namely the subject-specific extreme positions were obtained and kept successfully for the CT-scan protocol.

**Table 1:** Subjective specific angles (degrees, radio-ulnar deviation/flexion-extension/rotation) in six extreme positions

Subject	Radial deviation	Ulnar deviation	Dorsal extension	Palmar flexion	Pronation	Supination
1	18R*/11F/2	**30U**/3E/9	20R/**30E**/-3**	5R/**29F**/9	5U/8F/**-6**	14U/22E/**121**
2	**34R**/7E/8	**17U**/15E/20	21R/**74E**/15	9R/**15F**/13	16R/2E**/47**	3U/25E/**104**
3	**7R**/8E/11	**30U**/25E/13	25R**/74E**/10	4U/**25F**/19	10U/2F/**20**	29U/58E/**125**
4	**19R**/2F/10	**18U**/7E/17	4R/**63E**/8	11U/**42F**/20	2R/2F/**-20**	8U/34E/**128**
5	**23R**/1E/-12	**30U/**0E/0	19R/**70E**/-9	10R/**48F**/10	8U/8F/**-12**	30R/0F/**134**
6	**28R**/14F/-38	**35U**/13E/-11	16R/**50E**/-34	9U/**42F**/-4	10U/11F-16	30R/21E/**107**
7	**24R**/8F/16	**26U**/14F/24	8U**/47E**/16	10U/**47F**/19	13U/1F/**22**	4U/25E/**115**
8	**28R**/13F/-8	**34U**/16E/22	11U/**55E**/4	16U/**57F**/24	15U/5F/**-21**	20R/19E/**118**

*Bold means the target position, R means radial deviation, U means ulnar deviation, F means palmar flexion, E means dorsal extension. **minus means angle apex in the palmar side

Amidst six extreme wrist positions, area of the non-contact region in extreme dorsal extension (59.81 ± 26.46 mm^2^) was significantly the smallest, and it in extreme palmar flexion significantly was largest (170.51 ± 30.44 mm^2^). In these two positions, all subject could keep a little variance in the accompanied motions of the radio-ulnar deviation and the rotation.

The non-contact regions of the extreme radio-ulnar deviations were 113.73 ± 26.14mm^2^ and 103.69 ± 30.49 mm^2^, respectively. No significant difference was calculated in them. The accompanied motions seemed randomly and at will of the subject.

The non-contact regions of the extreme pronation and supination were 119.35 ± 52.50mm^2^ and 91.88 ± 22.17 mm^2^, respectively. No significant difference was calculated in them. The accompanied motions seemed randomly and at will of the subject.

The non-contact regions of six experimental extreme wrist positions increased in order of dorsal extension, supination, ulnar deviation, radial deviation, pronation and palmar flexion. As for two-group comparison, the non-contact region showed significantly larger (*p* < 0.05) in palmar flexion than the others except for in pronation, and in radial deviation (*p* < 0.05) than in dorsal extension (Tables 2 and 3).

**Table 2:** Non-contact areas of proximal scaphoid (mm^2^) in six extreme positions

Subject	Radial deviation	Ulnar deviation	Dorsal extension	Palmar flexion	Pronation	Supination
1	99.62	74.51	71.02	172.33	115.56	59.1
2	97.91	71.23	46.2	142.11	33.37	106.75
3	165.69	115.94	54.65	207.45	176.34	112.26
4	130.61	127.1	9.2	220.93	179.85	114.83
5	77.69	127.75	78.91	160.48	74.66	60.23
6	107.16	84.46	74.42	153.45	103.96	84.85
7	114.55	77.33	48.33	173.84	102.23	92.85
8	116.62	151.22	95.77	133.52	168.84	104.19
Mean	113.73***	103.69	59.81*	170.51*	119.35**	91.88
SD	26.15	30.49	26.46	30.44	52.50	22.17

*Area of the non-contact region in extreme dorsal extension (59.81 ± 26.46 mm2) was significantly the smallest (*p* < 0.05), and it in extreme palmar flexion (170.51 ± 30.44 mm2) significantly was largest (*p* < 0.05).The non-contact region showed significantly larger (*p* < 0.05) in palmar flexion than the others except for in pronation**, and in radial deviation *** (*p* < 0.05) than in dorsal extension

**Table 3:**
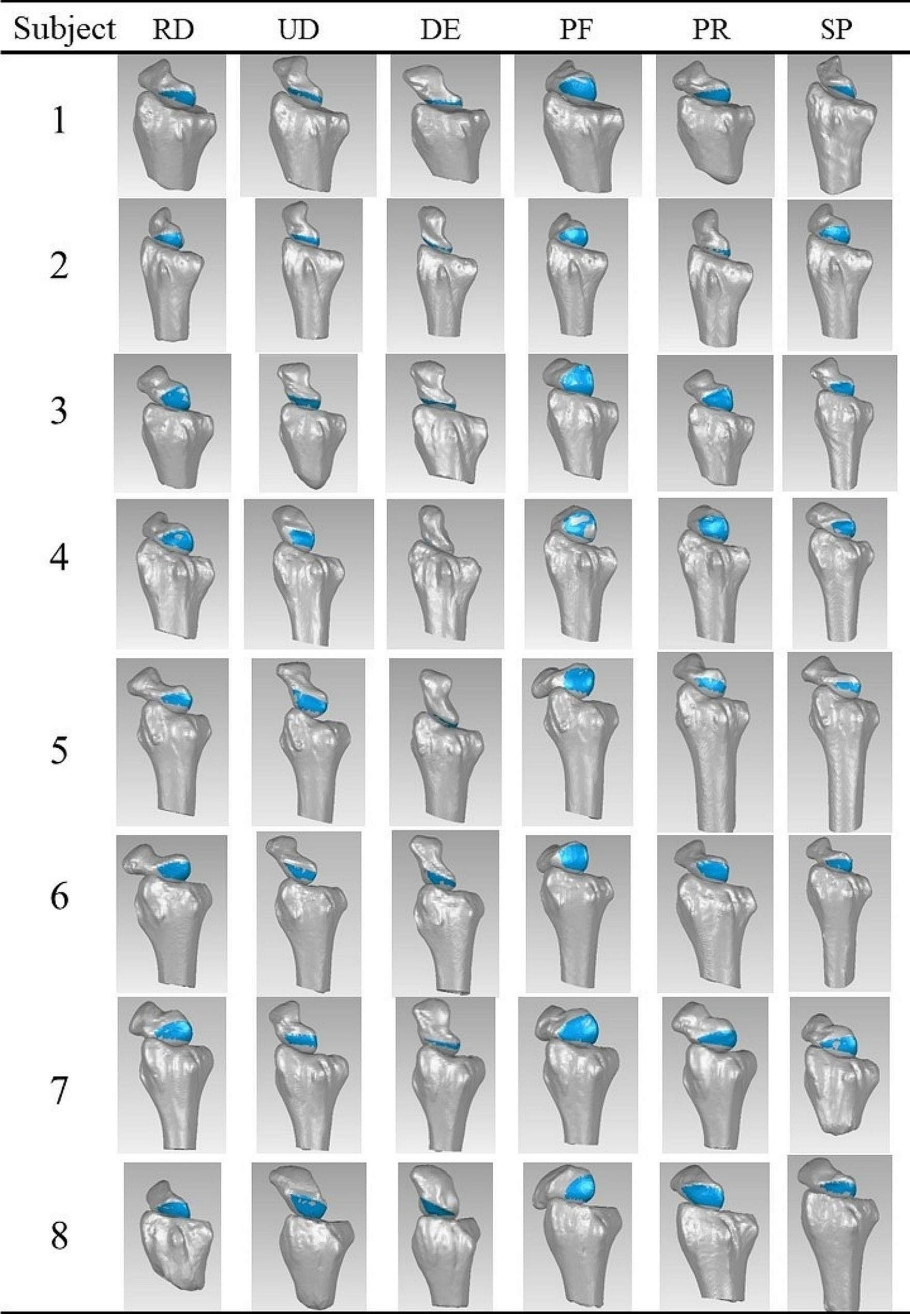
The key stage of volunteer’s six extreme wrist positions proceeding in Geomagic Studio

RD = radial deviation, UD = ulnar deviation, DE = dorsal extension, PF = palmar flexion, PR = pronation, SP = Supination, blue area means the non-contact region

Considering the subject-specific characteristic of the carpal bone, we calculated the percentage of the non-contact region over the whole surface area of the scaphoid (Table 4). The tendency of changes amidst different positions was the same as the real value (not the percentage).

**Table 4:** Non-contact areas in six extreme positions of proximal scaphoid over the area of whole scaphoid appearance (%)

Subject	Radial deviation	Ulnar deviation	Dorsal extension	Palmar flexion	Pronation	Supination
1	7.23	5.33	5.10	12.43	8.36	4.10
2	7.72	5.67	3.76	11.42	2.68	8.55
3	11.80	8.14	3.79	14.41	12.38	7.77
4	10.59	10.01	0.72	17.03	14.18	9.06
5	6.48	10.49	6.52	13.25	6.17	5.00
6	10.59	8.32	7.33	15.12	10.15	8.39
7	10.82	7.43	4.56	16.77	9.69	8.82
8	9.71	12.67	7.84	10.94	13.86	8.66
Mean	9.37	8.51	4.95	13.92	9.68	7.54
SD	01.96	2.48	2.31	2.31	3.93	1.90

## Discussion

Fractures of the scaphoid are the most common carpal injuries, representing 80-90% of all carpal fractures. The reported incidence of scaphoid fractures is usually over 10 per 100,000 person-years in the general populations [14–16]. There still occur about 5-15% nonunion of scaphoid fractures even with adequate primary treatment, which mainly results from the vulnerable blood supply of the proximal scaphoid and probably progresses to osteoarthritic changes several decades later [17]. To avoid this sequela, researches regarding to in vitro and in vivo scaphoid physiological characteristic and kinds of trials in clinical practice are being kept on going. With articular cartilage covering most appearance and no periosteum producing membrane bone, the fractured scaphoids are integrated only through the primary bone healing mode. Rigid fixation plays a key role in this healing process. Longer and stouter cannulated screw, implant perpendicular to the fracture plane as possible, and two screws would contribute much biomechanical stability, while synthesizing fragments of the scaphoid. Volar and dorsal approaches are usually performed to reduce the fragments and to insert the screws. Pros and cons swapping, dorsal approach is easy for optimal screw insertion [18], and volar approach is available for conservation of the blood supply. With the advancing wrist arthroscopy, 3D-print patient-specific drill guide, and intraoperative fluroscopic guidance, dorsal approach (mini-invasive and percutaneous technique) is being popular, through which we can implant the screw of more coincidence with biomechanics and with less disturbing tenuous blood supply of the scaphoid. Investigating the noncontact area of the dorsal proximal scaphoid in different wrist positions can facilitate preoperatively estimating insert point of the screw.

The scaphoid is one of the proximal row intercalated carpal bones, of which the geometry of the articular surfaces and interosseous ligaments control adaptive motions with forces from the subjective motions of the hand and wrist [19]. Considerable motion of the scaphoid occurs along the radius and the capitate. In extreme palmar flexion, the scaphoid flexed and deviated ulnarly give much more proximal articular surface out of the radial rim, the non-contact regions of proximal scaphoid as we called. In extreme dorsal extension, the scaphoid extended and rotated makes its proximal part hided into the radial fossa, which results in much lesser non-contact regions. Our measurements seemed to concur with the data of Rainbow [20]. No significant change were found of non-contact regions in the other four extreme positions. During two-group comparison, the non-contact region showed significantly larger in palmar flexion than the others except for in pronation individually, and in radial deviation than in dorsal extension. These data illustrated that radial deviation and pronation have a tendency to force the proximal scaphoid out of the radial rim, which appears no obviousness while combined with any other reverse-effect motions.

Area of the non-contact region in extreme dorsal extension (59.81 ± 26.46 mm^2^) was the smallest in our six extreme positions. The commercial cannulated screw for scaphoid fixation usually ends with outer diameter of around 3.0 mm, namely with area of 7.07mm^2^. These shall suggest there be sufficient non-contact region for one or two screws to insert from the dorsal approach in the selective scaphoid fractures. Our data shall give another information, scaphoid fractures except for very proximal broken could perform the dorsal transient percutaneous fixation with a k-wire at any position available for the manipulative reduction assisted with the X-rays, other than subjective flexion and pronation.

The extremely wrist motion of each participant (experimental volunteer) is not the same, owing to individual difference. And we should consider about this specificity before surgery. The perioperative surgical protocol including the measurement and calculation with medical analysis softwares, such as Mimics, Geomagic studio and 3ds-Max, may facilitate the surgical accuracy and efficiency. As for scaphoid fracture, only planes of the pre- or intra-operative X-rays cannot reveal the real fragment reduction or implant position. We should stay up-to-date, preoperative 3D analysis in how to reduce the fragments and where to insert the screws, and integrating with the intraoperative findings with wrist arthroscopy or fluoroscan to enhance the accuracy and to decrease the traditional open surgical damage to protect the scaphoid tenuous blood supply. Next, we would biomechanically analyze the different trajectory of the screws implanted with the guide wire through the non-contact area of the scaphoid proximal surface.

A limitation of our study is that we didn’t use posture devices to make uniform positions in a targeted position and the other two combined positions for all subjects, which might disturb the statistical results. In fact, our primary intention was to investigate the patient-specific wrist motions mimicking clinical diversity, we usually use joystick technique and maneuver the wrist to keep the scaphoid reduction in proper position other than the universal position, and the BMIs (Body Mass Index) might give some difference although with a fixed posture device.

We created fours planes to confine the proximal scaphoid to calculate its area as the non-contact data for analysis, based on anatomic markers on the scaphoid and radius, such as the radio-scaphoid articular line, radial styloid, sigmoid notch and the interosseous ligament attachment (dorsal scaphoid ridge). These subjective conception seemed pragmatic for the safe and accessible dorsal approach other than the name of non-contact region.

We concluded that sufficient space was available for the screw started from the dorsal approach despite the wrist positions.

The limitation of this study would be the small sample size and only the male and medical staff and single age-span group.

## Data Availability

All data generated or analyzed during this study are included in this published article, and the raw de-identified data may be made available upon reasonable request from the corresponding authors.

## Abbreviations

CT: Computed Tomography
ASCII: American Standard Code for Information Interchange
STL: Stereolithography
ECU: extensor carpi ulnaris tendon
BMI: Body Mass Index
